# Power Spectral Differences between Transient Epileptic and Global Amnesia: An eLORETA Quantitative EEG Study

**DOI:** 10.3390/brainsci10090613

**Published:** 2020-09-06

**Authors:** Jacopo Lanzone, Claudio Imperatori, Giovanni Assenza, Lorenzo Ricci, Benedetto Farina, Vincenzo Di Lazzaro, Mario Tombini

**Affiliations:** 1 Neurology, Neurophysiology and Neurobiology Unit, Department of Medicine, Università Campus Bio-Medico di Roma, 00128 Rome, Italy; g.assenza@unicampus.it (G.A.); lorenzo.ricci@unicampus.it (L.R.); v.dilazzaro@unicampus.it (V.D.L.); m.tombini@unicampus.it (M.T.); 2 Cognitive and Clinical Psychology Laboratory, Department of Human Sciences, European University of Rome, Via degli Aldobrandeschi 190, 00163 Rome, Italy; claudio.imperatori@unier.it (C.I.); benedetto.farina@unier.it (B.F.)

**Keywords:** qEEG, epilepsy, TGA, transient epileptic amnesia, EEG, LORETA, source imaging

## Abstract

Transient epileptic amnesia (TEA) is a rare epileptic condition, often confused with transient global amnesia (TGA). In a real-life scenario, differential diagnosis between these two conditions can be hard. In this study we use power spectral analysis empowered by exact Low Resolution Brain Electromagnetic Tomography (eLORETA) to evidence the differences between TEA and TGA. Fifteen patients affected by TEA (64.2 ± 5.2 y.o.; 11 female/4 male; 10 left and 5 right temporal epileptic focus) and 15 patients affected by TGA (65.8 ± 7.2 y.o.; 11 females/4 males) were retrospectively identified in our clinical records. All patients recorded EEGs after symptoms offset. EEGs were analyzed with eLORETA to evidence power spectral contrast between the two conditions. We used an inverse problem solution to localize the source of spectral differences. We found a significant increase in beta band power over the affected hemisphere of TEA patients. Significant results corresponded to the uncus and para-hippocampal gyrus, respectively Brodmann’s Areas: 36, 35, 28, 34. We present original evidence of an increase in beta power in the affected hemisphere (AH) of TEA as compared to TGA. These differences involve key areas of the memory network located in the mesial temporal lobe. Spectral asymmetries could be used in the future to recognize cases of amnesia with a high risk of epilepsy.

## 1. Introduction

Acute amnesic disorders are a common cause of admission to the emergency room and, among these, Transient Global Amnesia (TGA) accounts for the vast majority of cases [1].

However, a relevant group of patients with acute amnesia suffers instead from epilepsy with recurrent episodes and preserved consciousness [2]. This condition has been better defined and characterized as a syndrome named Transient Epileptic Amnesia (TEA) [3,4,5,6].

TEA is an insidious mimic of TGA. Missing the diagnosis means that seizures are likely to recur, leading to multiple emergency room visits and a linked social and economic burden [7].

In our previous paper, we retrospectively studied a population of 83 patients classified as TGA in the emergency room. After a complete diagnostic work-up, comprising standard EEG, 24 h EEG monitoring (24 h EEG) and 1.5 Tesla MRI, a consistent group (15/83) of these patients resulted to suffer from TEA [8], according to the most recent diagnostic criteria [4]. In a majority of TEA cases there was a delay of up to several months in the diagnosis, as no epileptiform activity was recognizable in the initial EEG. We concluded that TEA is challenging to diagnose, both from a clinical and neurophysiological perspective, and that standard EEG is often not conclusive.

Power Spectral Analysis (PSA) could aid in raising the neurophysiological suspect of an epileptic syndrome by showing alterations that can be missed with visual assessment [9]. These alterations usually involve quantitative oscillatory activity [9], which has a paramount role in many neurologic diseases [10].

In many epileptic conditions, previous papers have reported spectral alterations in the Affected Hemisphere (AH) of subjects suffering from temporal lobe epilepsy [11,12]. Meanwhile, TGA patients usually present a normal EEG. According to a recent study, only a small sub-group (about 26%) might show some non-specific acute abnormalities such as bilateral slowing [13]. Other studies on EEG spectral analysis in TGA patients found a diffuse symmetric decrease of beta and theta activity during the acute phase [14]. Meanwhile, there are no quantitative EEG (qEEG) studies describing spectral activity in patients with Transient Epileptic Amnesia.

The aim of the present study was to evidence the qEEG features of the interictal EEG of TEA and TGA patients, looking for spectral findings that differentiate the two conditions.

To achieve this goal, we used PSA empowered by exact low-resolution brain electromagnetic tomography (eLORETA) of standard clinical EEGs [15] to study spectral features of the EEG of patients with TEA compared to those with TGA. The use of source reconstruction techniques allowed us to project spectral activity on a brain template, allowing us to reach a deeper functional interpretation.

## 2. Materials and Methods

### 2.1. Study Population

**Figure 1:**
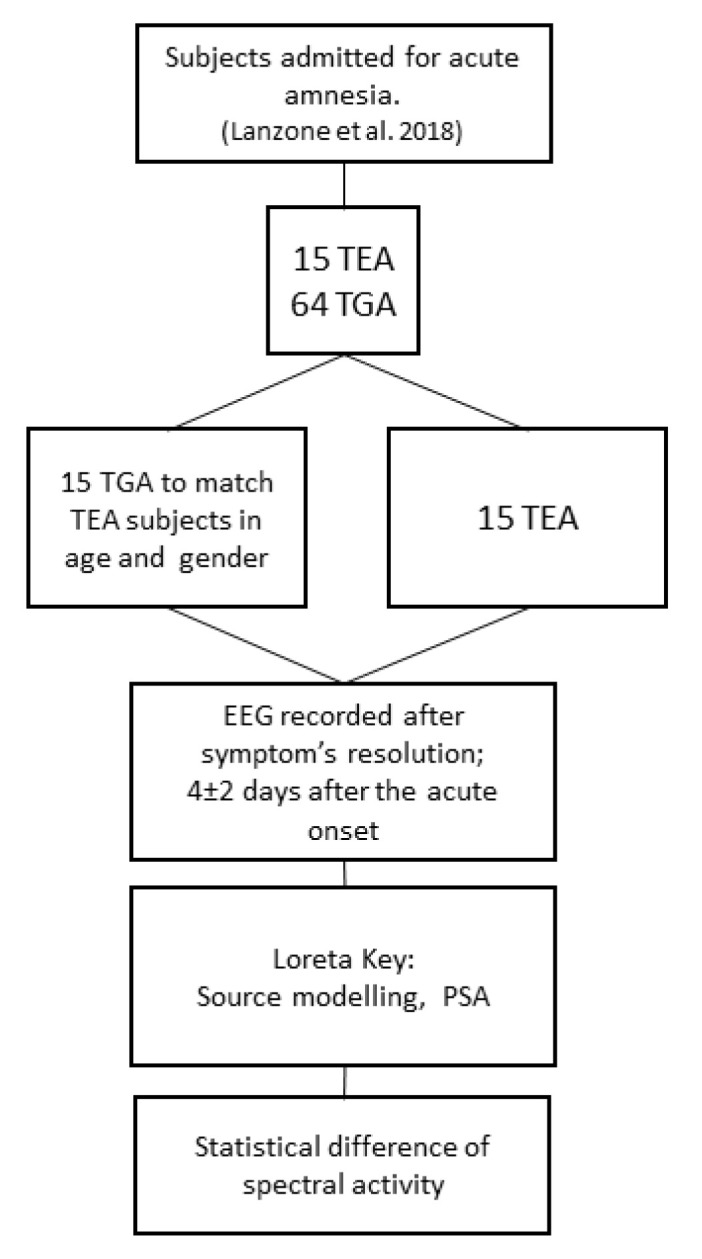
Depicts the flow chart of the study. TEA (Transient Epileptic Amnesia); TGA (Transient Global Amnesia).

We retrospectively identified 15 patients with TEA (mean age 67.2 ± 5.2 years; females/males: 11/4; 10 left and 5 right temporal epileptic focus) and 15 control patients affected by TGA, matched for age and gender, (mean age 65.8 ± 7.2 years, females/males: 11/4). Figure 1 depicts the flow chart of the study. Clinical features of patients with TEA were described in Lanzone et al. 2018, patients with TGA were randomly chosen among 68 patients from the previous study to be matched for age and sex and to meet the EEG criteria.

In both groups, EEG was recorded after the complete resolution of symptoms (on average 4 ± 2 days after the acute onset); all subjects were asymptomatic and fully recovered the anterograde and retrograde amnesia at the time of the EEG recording. Demographic data and instrumental findings from the two groups are reported in Table 1. More information on the TEA group is reported in Supplementary Table S1.

Inclusion criteria for the study were: (i) definite diagnosis of Transient Epileptic Amnesia/TGA (as defined respectively by Zeman et al. 1998 or Hodges and Warlow 1990); and (ii) EEG recorded during interictal period after the off-set of clinical symptoms (patient oriented in time and space and not symptomatic for amnesia, confusion or other neurological deficits).

Exclusion criteria were: (i) no focal lateralizing features at EEG or 24 h EEG (for the TEA group), impossibility to recognize the AH; (ii) other previous neurological diseases known to alter EEG rhythms (stroke, dementia, etc.) for both TEA and TGA groups; (iii) the absence of at least 180 sec of clean continuous eyes-closed EEG recording (for both groups); (iv) therapy with antiepileptic drugs (AEDs) or with drugs known to alter EEG (i.e., antidepressants, benzodiazepines); (v) all TEA patients had to be Drug Naïve at the moment of the EEG.

Elimination criteria: (i) constant artefactual EEG activity that could not be removed by preprocessing; (ii) more than one bad EEG channel (artefactual or high impedance).

Diagnosis of TEA was made according to the work from Zeman et al. 1998 [3], when the following criteria were fulfilled: (1) recurrent witnessed episodes of transient amnesia; (2) cognitive functions other than memory judged to be intact during typical episodes by a reliable witness; and (3) evidence for a diagnosis of epilepsy on the basis of one or more defined characteristics (3a: epileptiform abnormalities on electroencephalography; 3b: concurrent onset of other clinical features of epilepsy such as lip-smacking or olfactory hallucinations; 3c: clear cut response to anticonvulsant therapy).

In detail, all patients with TEA fulfilled criteria 1 and 2 and at least two of the supportive criteria as specified at point 3 of the Zeman’s criteria: 10 out of 15 patients had all 3 criteria (3a, 3b, 3c), 2 out of 15 had only criteria 3a and 3b (their outcome was not known as they were lost at follow-up) and 3 out of 15 patients had only 3a and 3c (no clear epileptic semiology was evident at history collection).

All patients underwent standard EEG, 24 h EEG and 1.5 Tesla MRI; MRI findings were noted and classified by a radiologist: patients were reported as having an altered MRI (such as gliosis or venous malformations; minor gliosis compatible with the age of the subjects was not considered as an alteration), DWI hippocampal alterations, typical finding in TGA [16], or no abnormal findings.

### 2.2. EEG Recordings

All patients underwent 19 channels EEG recording with standard 10–20 montage [17]; recordings were performed during quiet wake with eyes closed. Signal was recorded with a 32 channel Micromed device (SystemPlus software; Micromed, Mogliano Veneto, Italy). Impedance was kept below 5 KΩ. Reference electrode placed on the right mastoid bone, sampling rate was 256 Hz; A/D conversion was made at 16 bit; pre-amplifiers amplitude range was ±3200 µV [18]. The following hardware filters were used: high frequency filters (HFF) = 0.2 Hz. Signal was reviewed for referral by a neurologist experienced in epilepsy and EEG (M.T.). In patients with TEA lateralization was defined according to focal interictal epileptiform activity, amnestic history and clinical features. Qualitative visual evaluation of the EEG recordings showed no relevant evidence of sleepiness both in the TEA and the TGA group.

### 2.3. EEG Pipeline Analysis

EEG raw signal, 19 channels with mastoid reference, were imported in Matlab 2018 (Mathworks, Inc., USA). EEG signal was processed through EEGlab’s GUI for Matlab (Swartz Center for Computational Neuroscience, USA) [19] using the following pipeline.

(1) We selected 180 s continuous epochs of closed-eyed EEG for each subject and visually rejected macroscopic artefacts obtaining tracks of average 158 ± 13 s. Notch (50Hz), low-pass (70 Hz) and high-pass (0.5 Hz) EEG lab FIR (Finite Impulse Response) filters were applied to the EEG signal. (2) Interictal epileptiform abnormalities previously marked for removal by an experienced epileptologist (M.T.) were removed from quantitative EEG analysis; thus, a total of 13 suspected/epileptic discharges were rejected. (3) We performed an Independent Component Analysis (ICA) on the EEG using RUNICA script from Matlab (EEGLAB) [19] in order to remove blink and eye movement components (4). In the TEA group we transposed EEG matrix information of the 5 patients with right temporal epileptic focus so that all affected hemispheres (AH) resulted to be on the left. Hence in TEA group, the AH is on the left and the UH is on the right. (6) Data were exported for eLORETA Key 2019 [15,20,21,22] using a Matlab native script.

**Figure 2:**
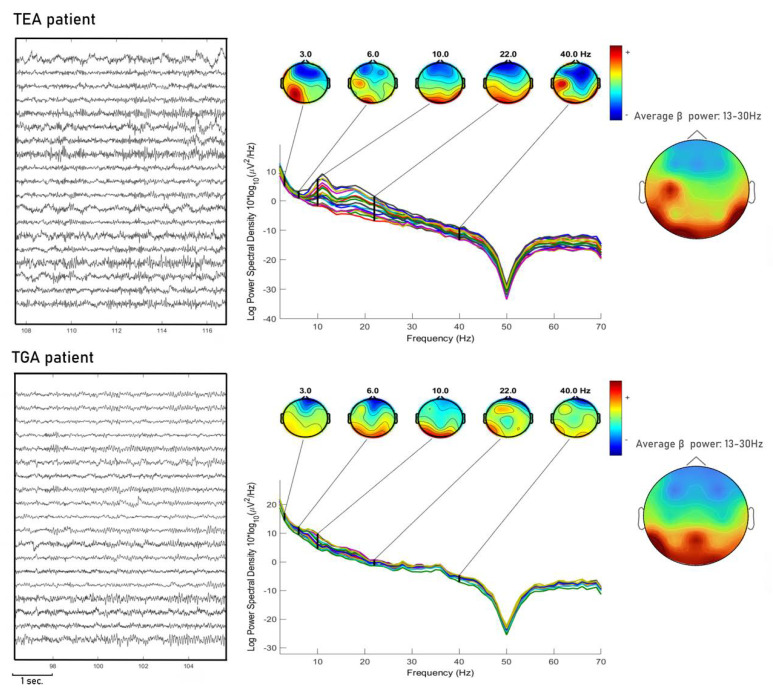
We show one exemplary subject for each condition TEA and TGA, PSA is shown with topographies of instantaneous frequency at each band (delta, theta, alpha, beta, gamma). Moreover, for each condition we show the topography in average beta power (13–30 Hz). Spectral activity in the TEA patient is more scattered and beta power topography appears to be more asymmetric compared to TGA patients.

In Figure 2 we show an example of EEG PSA.

In eLORETA, power spectral analysis was performed using Fast Fourier Transform algorithm, with a 2 s interval on the EEG signal. Spectral power was calculated for the following power bands in all scalp locations: delta (0.5–4 Hz), theta (4.5–7.5), alpha (8–12.5 Hz), beta (13–30 Hz) and gamma (30.5–60 HZ).

Topographic sources of EEG activities were determined using the LORETA algorithm. The computational task is to select the smoothest 3-dimensional current distribution [23,24], providing a true 3-dimensional tomography, in which the localization of brain signals is conserved with a low amount of dispersion [15].

The current eLORETA implementation uses a three-shell spherical head model registered to a standardized stereotactic space available as a digitized MRI from the Brain Imaging Centre Montreal Neurological Institute (MNI). EEG electrode coordinates are achieved using cross-registrations between spherical and realistic head geometry. In the current implementation, a spatial resolution of 7 mm is used, producing a total of 6239 voxels. eLORETA software manages to reconstruct low resolution electric tomography using EEG data finding a constrained solution to the EEG inverse problem [20,25].

The main advantage of using a technique of source imaging such as LORETA is that it allows to reconstruct the activity from the 19 channels on an ideal brain surface, thus giving the chance to observe spectral activity from a more physiologic and functional point of view. Rather than estimating the power of the activity of each single electrode, we can project the activity on the brain surface and localize it to the respective Brodmann Areas (BAs).

We chose to use eLORETA since it is a thoroughly validated method for source localization (Pascual-Marqui et al. 2011), and has implemented non-parametric statistic to compare PSA, with in-built correction for multiple comparisons [26]. We used a 3-shell reconstruction model since it is more robust to segmentation and forward modelling errors as compared to Boundary Element Method in cases, as ours, where MRI volumetric images for each patient are not available [27]. We recognize that using source localization with 19 channels is not as accurate as when it is used with high definition EEG, nevertheless it was shown that even 19 channel montages allow good projection or scalp activity on a brain model and this method has already been validated [28,29,30,31].

### 2.4. Data Availability

RAW unprocessed data supporting the findings of this study are available from the corresponding author, upon reasonable request.

### 2.5. Statistics Analysis

Comparisons between clinical features of the two groups were made with Chi-squared test.

Comparisons in PSA (TEA versus TGA) were performed on log transformed data using the nonparametric statistical mapping methodology supplied by the eLORETA software [26]. This methodology is based on Fisher’s permutation test. LORETA’s inbuilt nonparametric randomization procedure was performed to take into account multiple comparisons. Significance thresholds were set by the software implemented in eLORETA.

T-level thresholds corresponding to statistically significant *p* values (*p* < 0.05) were calculated [32].

Correction of multiple comparisons was performed using the statistical nonparametric mapping (SnPM) methodology [26] included in the eLORETA program package (for more details see [33,34]). Briefly, this procedure computes 5000 data randomizations to determine the critical probability threshold of T-values [26] corresponding to a statistically corrected (i.e., after the multiple comparisons among all voxels in each frequencies) *p* values. Alpha was set to *p* = 0.05 [35]. We sorted voxel values according to the threshold computed by eLORETA (T = ±4.12) and corresponding to *p* = 0.05.

Furthermore, the eLORETA software provides effect size thresholds for t-statistics corresponding to Cohenʹs d values (Cohen, 1988): small = 0.2, medium = 0.5, large = 0.8. The effect sizes for the T-threshold were T = 1.058, T= 2.646, and T= 4.233, corresponding, respectively, to small, medium, and large effect sizes.

RAW voxel values from significant MNI coordinates were extrapolated manually and saved for further analysis, these data were confronted using U Mann–Whitney test.

Since five TEA subjects presented a right temporal lobe epileptic focus, for the purpose of eLORETA analysis, we transposed their EEG matrix, thus assuming equivalence of comparisons in PSA between TEA AH (5 right and 10 left hemispheres) and TGA (all left hemisphere). We also tested spectral differences in MNI coordinates that resulted in being significant after randomizing (www.random.org) hemispheres of the TGA condition and testing them against AH and UH from TEA, this was done to avoid the confounder of testing all affected TEA hemispheres with only left TGA hemispheres.

## 3. Results

### 3.1. Clinical Features

Clinical features of both populations are described in Table 1. TEA and TGA showed, as expected, significant differences in: presence of interictal epileptiform abnormalities on the standard EEG (TEA 8/15, TGA 1/15; Chi-squared test, *p* = 0.005); interictal epileptiform abnormalities on wake 24 h EEG (TEA 10/15, TGA 1/15; Chi-squared test, *p* = 0.001); and interictal epileptiform abnormalities on sleep 24 h EEG (TEA 15/15, TGA 0/15; Chi-squared test, *p* = 0.001).

Other clinical features did not evidence significant differences: history of migraine (TEA 1/15, TGA 0/15; Chi-squared test > 0.05); obstructive sleep apnea syndrome (TEA 2/15, TGA 0/15; Chi-squared test > 0.05); patent foramen ovale (TEA 0/15, TGA 1/15; Chi-squared test > 0.05). No seizures were recorded during the experiments.

MRI findings showed no difference in frequency of MRI DWI hippocampal alterations (TEA 0/15, TGA 0/15; Chi-squared test, *p*= 0.309), and in MRI abnormal findings in the TEA group (TEA 5/15, TGA 1/15; Chi-squared test, *p* =0.068).

### 3.2. Power Spectral Differences

**Figure 3:**
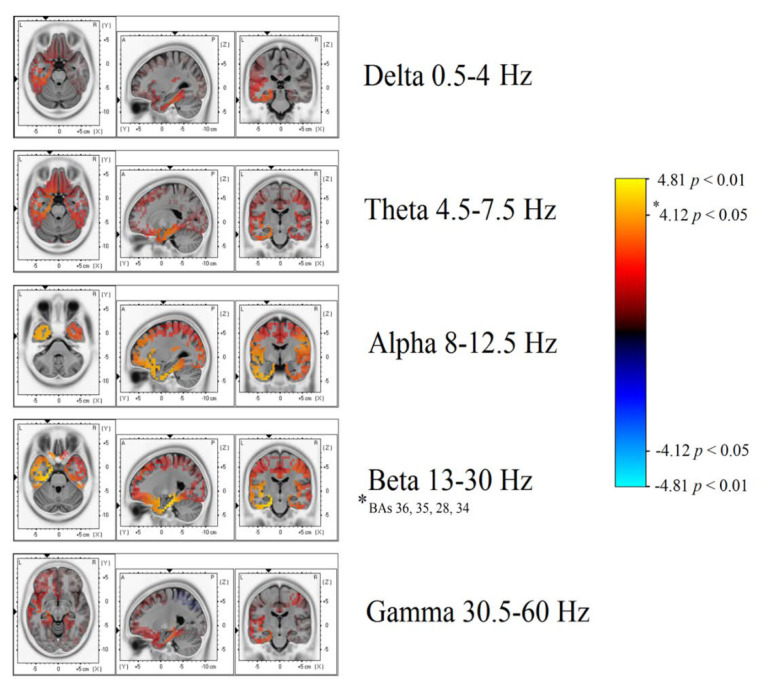
Depicts a general overview of power spectrum contrasts at various band widths, projected on a brain template as reconstructed by eLORETA’s inverse model.

**Figure 4:**
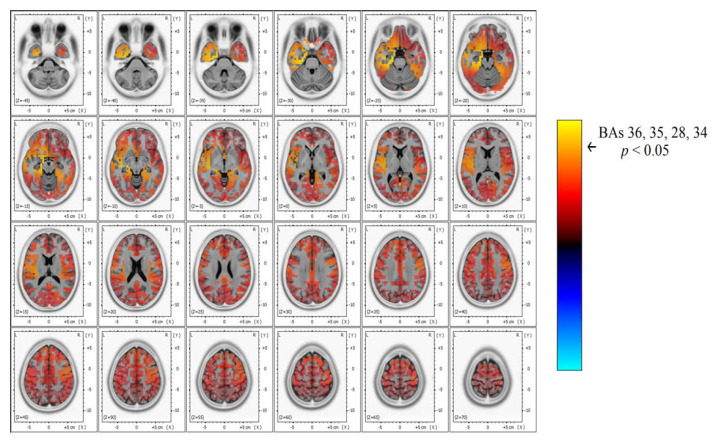
In-depth detail of power spectrum contrast in the beta band width, showing a significant power increase (yellow) over the left temporal area that corresponds to TEA subjects’ AH compared to TGA.

Significant differences in beta band power were found, showing increased power in the AH of patients with TEA when compared to the TGA group (Figure 3 and Figure 4). Using the inverse problem solution empowered by eLORETA allowed us to localize this power difference to the para-hippocampal gyrus (Brodmann’s Area, BAs 36-35-28-34, *p* < 0.05) and the uncus (BA 28, *p* < 0.05) (Table 2). Significant differences in beta power comparing TEA and TGA, showing increased Beta power in the AH of TEA patients, were found in the following regions of interest: AH, Parahippocampal gyrus (l); BA 36, T = 4.20; *p* = 0.042; AH, Parahippocampal gyrus (l); BA 35, T = 4.20; *p* = 0.042; AH, Parahippocampal gyrus (l); BA 28, T = 4.18; *p* = 0.044;AH, Uncus (l); BA 28, T = 4.14; *p* = 0.047; AH, Parahippocampal gyrus (l); BA 34, T = 4.12 *p* = 0.049.

No significant difference in beta power was found in the unaffected hemisphere (UH).

No significant differences were observed in the other frequency bands: Theta, Alpha, and Delta.

A statistical trend towards increased power was observed in the alpha frequency band in the left AH, uncus (BAs 20-28, *p* = 0.08). AH, uncus (l); BA 20, T = 3.83; *p* = 0.080; AH, Uncus (l); BA 28, T = 3.83, *p* = 0.080. Detailed eLORETA statistics are reported in Table 2.

U Mann–Whitney test confirmed significant differences (*p* < 0.05) in all MNI coordinates when confronting AH with randomized TGA hemispheres (random 5 right hemispheres and 10 left) showing an increase in beta power in the AH of TEA patients: BA 28 (U= 4.4, *p* = 0.035), BA 35 (U = 5.06, *p* = 0.03), BA 36 (U = 5.4, *p* = 0.028), BA 34 (U = 4.18, *p* = 0.044).

## 4. Discussion

Our study shows, for the first time, interictal spectral differences when comparing the EEG of TEA and TGA subjects. PSA statistical difference map evidenced an asymmetric increase in Beta band power over the AH of patients with TEA. Through a LORETA inverse problem solution, we were able to localize spectral changes (Figure 3 and Figure 4) to the temporal lobe of the AH, more precisely to the uncus and parahippocampal gyrus (Table 2 for specific BAs).

We found strong evidence of altered oscillatory activity in areas located in the mesial temporal lobe of the AH and involved in functions such as episodic memory and recall [36]. Although the accuracy of source reconstruction with 19 electrode caps is lower compared to more dense EEG caps [37], we believe that the neurophysiological alterations we found could relate to memory impairment in epilepsy [38,39] and in particular in TEA [3,4,5].

We can infer that these differences are generated by background activity and not by ictal/interictal activity, since epileptic discharges were manually removed from the EEGs of TEA patients. The EEGs of TGA patients were assumed to be comparable to that of healthy subjects since symptoms were fully regressed at the time of recording [40], and no particular findings were reported in the EEG’s referral.

Our results, thus, suggest that this alteration in beta oscillatory activity could depend on brain dysrhythmic activity related to the chronic epileptic condition of TEA subjects.

The present study is the first to demonstrate spectral differences when comparing interictal EEG from patients with TEA and TGA, two conditions that are usually hard to tell apart [8].

### 4.1. Neurophysiological Role of Beta Power Increase in Transient Epileptic Amnesia

Literature on EEG spectral features in epilepsy usually report an increase in the delta and theta band over the AH [12]. In our study we did not find significant alterations in low-frequency bands. We hypothesize that low-frequency spectral alterations are caused by the slow wave component of interictal epileptic activity, that, in our case, was manually removed [41,42]. Meanwhile there are no reports in the literature that link an increase in alpha/beta activity to epileptic interictal activity.

Hence, we propose that alteration in the beta band over the AH of TEA subjects may be related to background, non-paroxysmal, temporal lobe disfunction. These findings are likely a sign of temporal dysrhythmic activity, caused by network disruption in the context of a withstanding epileptic condition. To support this hypothesis, many recent studies show changes of large scale and local network connectivity in focal epilepsy [43,44,45].

Further studies are needed to conclude whether this beta power alteration over the AH is directly pathological or rather a compensatory increase to keep up with memory tasks in a pathologic network. This second hypothesis could be supported by the fact that beta and alpha oscillations are known to have a role in working memory encoding tasks and in memory consolidation [46,47,48,49].

### 4.2. Neurophysiological Disfunction and Accelerated Long-Time Forgetfulness

Patients with TEA are often described to suffer from a peculiar form of chronic memory impairment: Accelerated Long-time Forgetfulness (ALF) [50]. ALF was described in these patients as a peculiar deficit in memory fixation [35,51,52], and its nature is not clear, but this phenomenon is probably related to the involvement of the temporo-mesial network that has a key role in memory fixation [53,54].

Recent functional-MRI (fMRI) findings point toward a temporal impairment in ALF. As confirmed by fMRI during memory recalling tasks, TEA patients show different activation patterns in the affected para-hippocampal cortex when compared to controls [55]. The authors hypothesized that ALF could be caused by a deficit of memory encoding networks induced by the epileptic focus. We strongly agree with this hypothesis, as our data are consistent with this theory and could be regarded as an evidence of neurophysiological alteration in the background activity of the affected temporal lobe in cases of TEA.

Unfortunately, due to the retrospective nature of the study, we could not test our patients for ALF, and thus beta alteration in the temporal lobe of TEA patients is yet to be directly linked with ALF.

### 4.3. Potential Clinical Implications and Research Directions

A point of strength of our study is that patients with TEA were off medication at the time of the EEG recording. In fact, AEDs are known to alter connectivity and PSA [56,57] and are considered a strong confounding factor for qEEG studies, often hard to avoid.

Our study is an additional proof of how qEEG might add value to the visual referral of EEG. We found quantitative differences between patients with TEA and TGA, these differences could be used in the future, eventually combined with other findings that characterize epilepsy such as heart rate variability [58] or blood work [59], to aid clinicians in differential diagnosis.

Further studies, including specific neuropsychological tests for ALF and a larger sample size, could clarify the role of neurophysiologic alterations in ALF and show the diagnostic yield of diagnostic algorithms that include qEEG features [60].

## 5. Limitations

Our study suffers from some limitations that need to be addressed. We used 19 channels of EEG due to the clinical nature of the study. LORETA source modelling has been used with good results with different brain imaging techniques and also with 19 channel EEGs [28,30], but it is due to say that the precision in localization is reduced if compared with modern high-definition EEGs.

It is noteworthy that in order to have the highest sensitivity in diagnosing epilepsy, patients should undergo prolonged video EEG monitoring, but this practice is expensive, time-consuming and often reserved for the most severe cases and presurgical evaluations.

We compared TEA with TGA, enrolling patients when asymptomatic, we assumed that after symptoms resolution, patients with TGA have normal EEG. This is supported by the fact that the literature describes EEG alterations in TGA exclusively in the acute symptomatic phase, and there are no reports on EEG after symptoms resolution. Nevertheless, further studies also including healthy subjects would add power to this finding.

Patients could not be tested for accelerated long-time forgetfulness or other neuropsychological tests since these tests are not part of routine practice and since the study design is retrospective.

Finally, due to the rarity of TEA, the numerosity of our sample is limited and not all patients enrolled shared the same focus side.

Following our study, larger studies could leverage differences in spectral activity to design differential diagnostic algorithms (TEA versus TGA) based on qEEG.

## 6. Conclusions

Our paper presents original evidence of increased interictal beta spectrum power in the AH of TEA patients when compared to TGA patients. These differences were localized to the AH uncus and para-hippocampal gyrus. Beta band alterations specific to the AH might be related to temporal network malfunctioning and could explain symptoms such as the ALF phenomenon observed in patients with TEA.

## Figures and Tables

**Table 1:** Clinical and neurophysiological data from the TEA and TGA groups. * MRI noted as “minor alteration” were four cases of nonspecific gliosis and one small, sub-centimetric cavernoma in the TEA group, and one case of gliosis in the TGA group.

Demographic	TEA (15)	TGA (15)
Age	67.2 ± 5.2	65.8 ± 7.2
Sex	11F 4M	11F 4M
Epileptic focus	10L/5R	-
**Comorbidities**		
Migraine	1 (6%)	0 (0%)
OSAS	0	2 (13%)
PFO	0	1 (6%)
**EEG Findings**		
standard EEG alteration	8 (53%)	1 (6%)
Holter EEG positivity	15 (100%)	0 (0%)
IEA Awake	10 (6.66%)	1 (6%)
IEA Sleep	15(100%)	0 (0%)
**MRI Findings**		
Minor MRI alteration *	5 (26.3%)	1 (6%)
Mesial sclerosis	0	0
Hippocampal DWI+	0	1(6%)

OSAS (Obstructive Sleep Apnea Syndrome); PFO (Patent Foramen Ovale); IEA (Interictal Epileptiform Abnormalities); TEA (Transient Epileptic Amnesia); TGA (Transient Global Amnesia).

**Table 2:** Detailed eLORETA statistics for the between group comparison (TEA vs. TGA). Significant differences as well as the most prominent modifications are reported for each frequency band.

eLORETA MNI Coordinates			Brain Structure	Brodmann Area	Frequency Band	T Value	*p* =
*x*	*y*	*z*					
−30	−30	−25	Parahippocampal gyrus (l)	36	Delta	2.62	0.499
−20	−20	−20	Parahippocampal gyrus (l)	28	Theta	3.05	0.278
−30	−5	−40	Uncus (l)	20	Alpha	3.83	0.080
−25	−5	−25	Uncus (l)	28	Alpha	3.83	0.080
−25	−20	−30	Parahippocampal gyrus (l)	36	Beta	4.20	0.042
−25	−20	−25	Parahippocampal gyrus (l)	35	Beta	4.20	0.042
−20	−15	−25	Parahippocampal gyrus (l)	28	Beta	4.18	0.044
−20	−10	−35	Uncus (l)	28	Beta	4.14	0.047
−20	−10	−20	Parahippocampal gyrus (l)	34	Beta	4.12	0.049
−25	−20	−10	Parahippocampal gyrus (l)	28	Gamma	2.63	0.493

Abbreviation: eLORETA = exact Low Resolution Electric Tomography software; MNI = Montreal Neurological Institute; (l) = left; (r) = right.

## Supplementary Materials

The following are available online at https://www.mdpi.com/2076-3425/10/9/613/s1: Supplementary Table S1: The table depicts clinical information on the amnesic episodes of patients with Transient Epileptic Amnesia, along with the therapy of choice started after the diagnosis.
