# Supplementary material for: Power Spectral Differences between Transient Epileptic and Global Amnesia: An eLORETA Quantitative EEG Study
Source: Brain Sci. 2020 Sep 6;10(9):613. doi: 10.3390/brainsci10090613 (PMC7563784; doi:10.3390/brainsci10090613)
Supplement: Supplementary file 1 [file brainsci-10-00613-s001.pdf]

Supplementary Table 1. The table depicts clinical information on the amnesic episodes of patients with Transient Epileptic Amnesia, along with therapy of choice started after the diagnosis.

Patient	Age	Sex	Focus	Duration (Hours)	Recurrence	Onset	Symptoms other than amnesia	Therapy of choice
1	69	F	Left	2,5	2	morning	Confusion	CBZ 400 mg
2	70	M	Right	3	5	morning (awakening)	Confusion	LEV 3000 mg
3	75	M	Left	12	3	morning (awakening)	Confusion	LEV 1000 mg
4	63	F	Left	6	2	morning	headache	LTG 200 mg
5	60	M	Left	0.4	3	afternoon	-	LEV 1500 mg
6	64	F	Right	1	2	morning	-	LTG 150 mg
7	67	F	Left	1	5	morning	language disorder	LEV 1500 mg
8	59	F	Left	5	4	morning (awakening)	confusion and headache	CBZ 800 mg
9	61	F	Left	2	2	morning	spatial disorientation	LEV 1000 mg
10	68	F	Right	24	5	morning	spatial disorientation	ZNS 200 mg
11	74	F	Right	1	3	morning (awakening)	spatial disorientation	LEV 1000 mg
12	66	F	Left	3	2	evening (awakening)	-	LEV 1250 mg
13	70	F	Left	4	2	morning (awakening)	post ictal confusion (2 days)	LEV 1000 mg
14	75	F	Left	5	2	afternoon	confusion aphasia	LEV 1000 mg
15	67	M	Right	2	2	-	confusion	LEV 1000 mg

CBZ (Carbamazepine); LEV (Levetiracetam); LTG (Lamotrigine)
